# Supplementary figures and images for: Genetic divergence between populations of feral and domestic forms of a mosquito disease vector assessed by transcriptomics
Source: PeerJ. 2015 Feb 26;3:e807. doi: 10.7717/peerj.807 (PMC4349049; doi:10.7717/peerj.807)

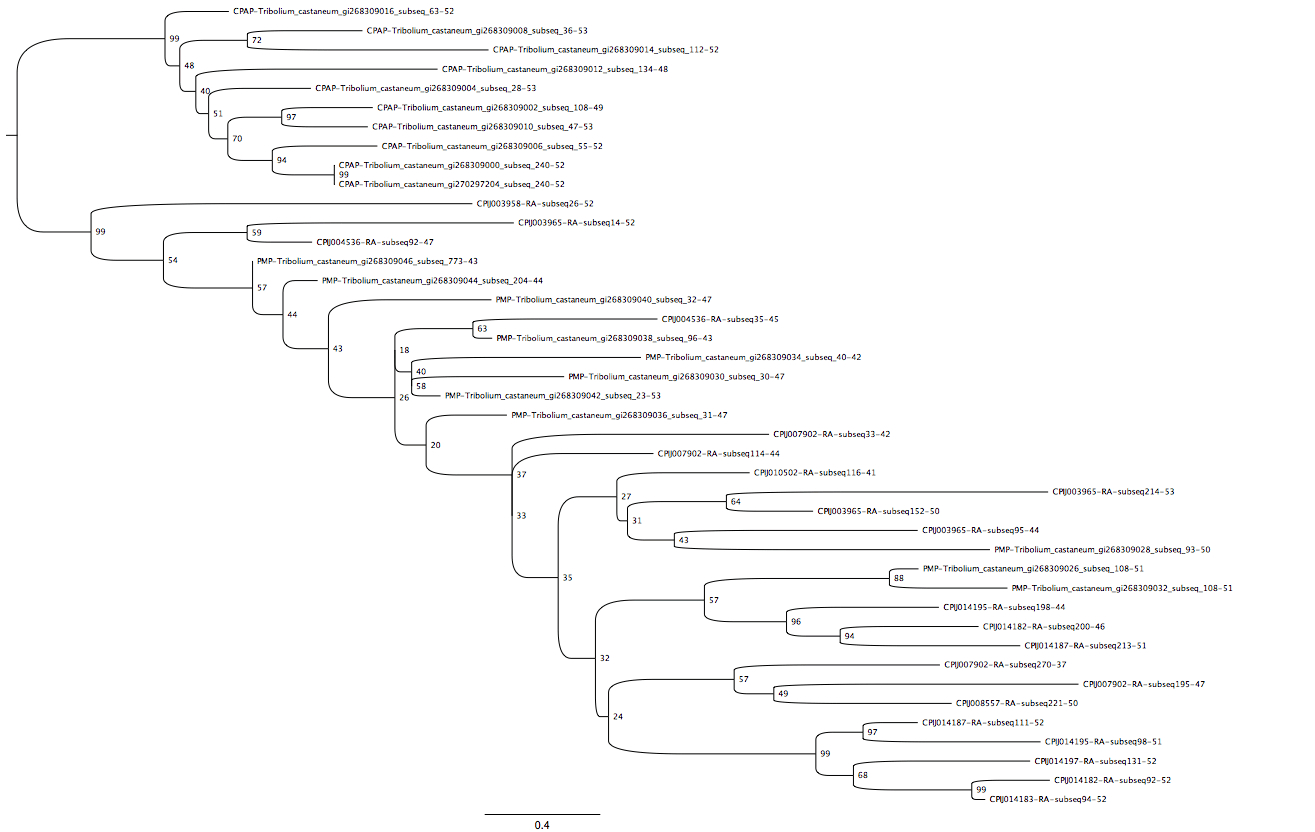

Supplement: Figure S1 — Maximum-likelihood phylogenetic tree showing monophyly of peritrophin-A domains reported here with peritrophic matrix proteins (labeled PMP), exclusive of the cuticular proteins analogous to peritrophins (labeled CPAP) of Jasrapuria et al. (2010). NCBI GI numbers are appended to Tribolium castaneum sequence IDs; all sequences are suffixed with “_subseq_[coordinate of first amino acid extracted]-[length of extracted peptide window]”. [file peerj-03-807-s001.jpg]
